# Low concentration of formononetin promotes proliferation of estrogen receptor-positive cells through an ERα-miR-375-PTEN-ERK1/2-bcl-2 pathway

**DOI:** 10.18632/oncotarget.21923

**Published:** 2017-10-19

**Authors:** Yan-Hong Guo, Feng-Yan Tang, Yong Wang, Wen-Jun Huang, Jing Tian, Hui-Ling Lu, Min Xin, Jian Chen

**Affiliations:** ^1^ Department of Physiology, Guilin Medical University, Guilin, China; ^2^ The First Clinical Medical School, Guilin Medical University, Guilin, China; ^3^ Department of Pathophysiology, Guilin Medical University, Guilin, China

**Keywords:** formononetin, ERK, apoptosis, miR-375, PTEN

## Abstract

A low dose of formononetin accelerates the proliferation of nasopharyngeal carcinoma cells *in vitro*; however, the underlying mechanism remains unknown. Here, we investigated the molecular mechanism of formononetin in CNE2 cell proliferation. CNE2 cells were treated with 0 to 1 μM formononetin. To inhibit mitogen activated protein kinase / extracellular regulate kinase (MAPK/ERK) kinase (MEK) and microRNA (miR)-375, cells were pretreated with either PD98059 or a miR-375 inhibitor, respectively, followed by co-treatment with formononetin (0.3 μM) plus an inhibitor. Female rats were ovariectomized (OVX), and some OVX rats received formononetin or estrogen (E_2_) injections. Sham operated animals were used as controls. Compared to control, 0.3 μM formononetin accelerated proliferation and decreased late apoptosis of CNE2 cells. However, formononetin-induced pro-growth and anti-apoptosis activity was abolished by PD98059 and the miR-375 inhibitor. In addition, 0.1 and 0.3 μM formononetin significantly increased estrogen receptor-α (ERα) and bcl-2, but decreased protein-phosphatase and tensin homologue (PTEN) protein expression, all of which was reversed by the miR-375 inhibitor. Additionally, formononetin treatment resulted in a transient upregulation of phosphorylated (p)-ERK1/2. *In vivo* studies indicated that formononetin significantly increased endometrium thickness and down-regulated ERα expression in OVX rats. Taken together, our study demonstrates that a low concentration of formononetin can promote growth of CNE2 cells and uterine tissues, possibly through regulating the ERα-miR-375-PTEN-ERK1/2-bcl-2 signaling pathway.

## INTRODUCTION

Nasopharyngeal carcinoma (NPC) is an endemic malignant disease of the head and neck. The etiology of NPC is complex and multifactorial, including genetic susceptibility, Epstein-Barr virus (EBV) infection, and exposure to chemical carcinogens [1]. Moreover, emerging evidence indicates that there is an abnormal up-regulation of the estrogen receptor (ER) in NPC tissues, which is associated with carcinogenesis and progression of NPC [2, 3]. *In vitro* work further demonstrated that the adhesion, migration, and invasive capabilities of NPC cells, HNE1, were enhanced by ER suppression [4]. These data imply a crucial role of ER in modulating the biological behaviors of NPC cells.

Formononetin, a typical phytoestrogen, is the main active component of red clover plants. Accumulating evidence indicates that formononetin possesses a variety of pharmacological effects, including antioxidant [5], anti-tumor [6–9], and anti-inflammatory [10] properties. Due to its structural similarity to estrogen, formononetin exerts several estrogen-like effects by binding to the ER and regulating gene expression [11, 12]. ERs are transcriptional factors that belong to the nuclear receptor superfamily. Two types of ERs have been identified, namely ERα and ERβ. ERα and ERβ can exert opposite actions in mediating cell growth. For instance, the proliferative effects of estrogen (E_2_) in the breast are attributed to ERα, while ERβ is thought to serve an anti-proliferative role in the presence of E_2_[13]. In addition, alteration of ERα by chemicals may alter the cell proliferation index [14].

MicroRNAs (miRNAs) have been implicated in the pathogenesis of cancer. Our previous study indicated that there is positive feedback regulation between ERα and miR-375 in breast cancer MCF-7 cells [15]. Protein-phosphatase and tensin homologue (PTEN), a tumor suppressor, has been found to control cell survival, proliferation, and apoptosis [16, 17]. Down-regulation of PTEN leads to tumor cell invasion and metastasis in NPC patients [18]. Other studies revealed that miRNAs promote growth and metastasis of NPC cells through suppressing PTEN expression [19, 20]. Nevertheless, the association between miR-375 and PTEN in NPC development has not been clarified.

Our previous study demonstrated that low concentrations of formononetin (< 0.3 μM) were capable of stimulating cell proliferation and inhibiting cell apoptosis in CNE2 cells by up-regulating bcl-2 and p-ERK1/2 expression [21]. This suggests that formononetin is potentially involved in an ER-MAPK/ERK-bcl-2 signaling pathway that promotes growth. In the present study, we investigated the effects of formononetin on ER and MAPK signaling in an ER-positive NPC cell line (CNE2) by pharmacologically inhibiting MAP2K1 with PD98059. Moreover, we measured the involvement of the miR-375-PTEN pathway in formononetin-treated CNE2 cells. In addition, ovariectomized (OVX) rats, which are deficient in endogenous estrogen, were used to investigate the effects of formononetin on ERα expression in uterine tissues *in vivo*.

## RESULTS

### Formononetin stimulates CNE2 cell proliferation

**Figure 1:**
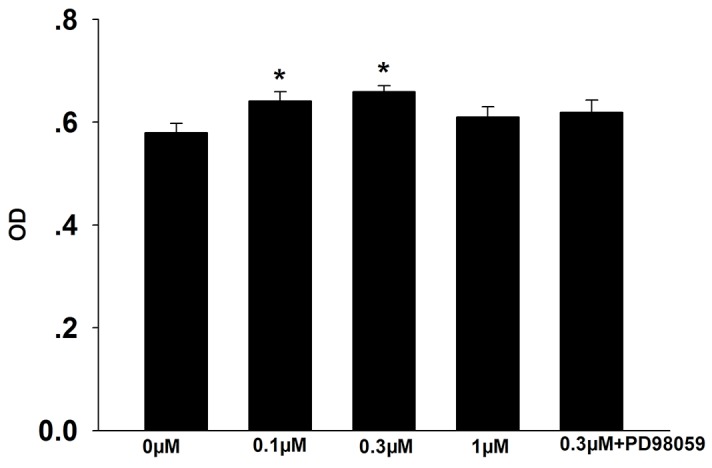
Effects of formononetin on CNE2 cell proliferation The number of CNE2 cell was significantly increased in 0.1 μM and 0.3 μM formononetin group but not in the group pretreated with PD98059. OD, optical density. ^*^ = P < 0.05 vs control; n=7.

Cell proliferation was evaluated using the MTT assay. As shown in Figure 1, treatment with 0.1 μM and 0.3 μM formononetin significantly increased the number of CNE2 cells compared to the control (control, 0.58 ± 0.02; 0.1 μM formononetin, 0.641 ± 0.018; 0.3 μM formononetin, 0.659 ± 0.012; p < 0.05). However, inhibition of the MEK signaling pathway using the MAP2K1 inhibitor, PD98059, reversed formononetin’s effect on cell proliferation (0.619 ± 0.02; p > 0.05 *vs*. control). 1 μM formononetin did not have an effect on proliferation (0.61 ± 0.02%; p > 0.05). These data suggest that formononetin, at a dose of 0.3 μM, stimulates proliferation in CNE2 cells, possibly through regulating the MEK signal pathway.

### Formononetin decreases apoptosis of CNE2 cells

**Figure 2:**
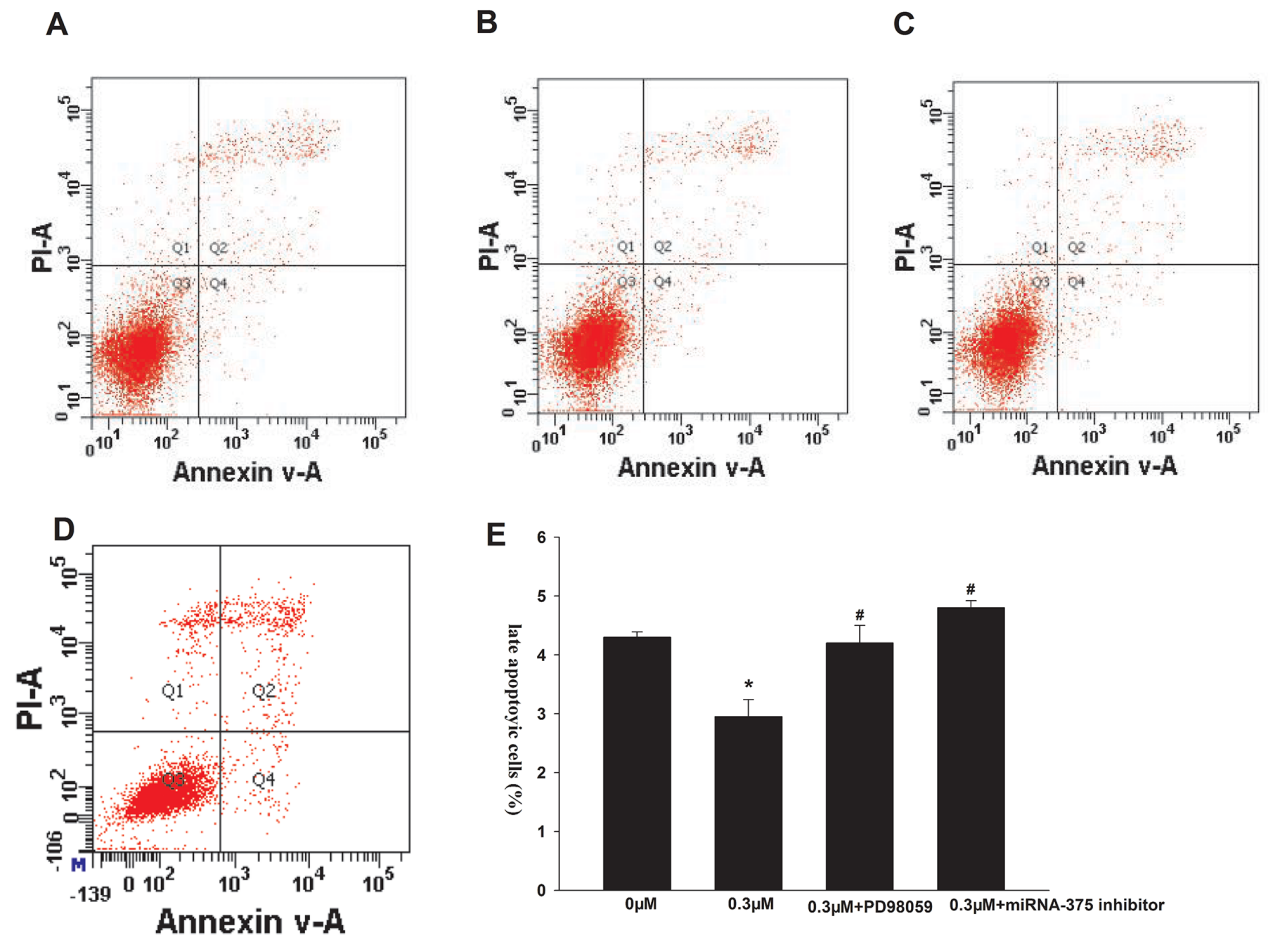
Effects of formononetin on the late apoptosis of CNE2 cells the late apoptosis of CNE2 cells were inhibited by 0.3 μM formononetin. When CNE2 cells were pretreated with PD98059, the rate of late apoptosis was similar with the control. **(A)** control group; **(B)** 0.3 μM formononetin group; **(C)** 0.3 μM formononetin group+PD98059; **(D)** 0.3 μM formononetin group+ miR-375 inhibitor and **(E)** rate of late apoptosis in CNE2 cells. ^*^=P < 0.05 vs control; n=3. ^#^=P < 0.05 vs 0.3 μM formononetin group; n=3.

We next investigated the effects of formononetin on cell apoptosis. As shown in Figure 2, incubation with 0.3 μM formononetin greatly reduced the percentage of late apoptotic cells compared to the control (control, 4.30 ± 0.09%; 0.3 μM formononetin, 2.95 ± 0.29%; p < 0.05). Administration of PD98059 or the miR-375 inhibitor abolished the formononetin-mediated inhibitory effect on late apoptosis (PD98059, 4.20 ± 0.3%; miR-375 inhibitor, 4.8 ± 0.12; p < 0.05 *vs*. 0.3 μM formononetin).

### Formononetin increases miR-375 mRNA expression in CNE2 cells

**Figure 3:**
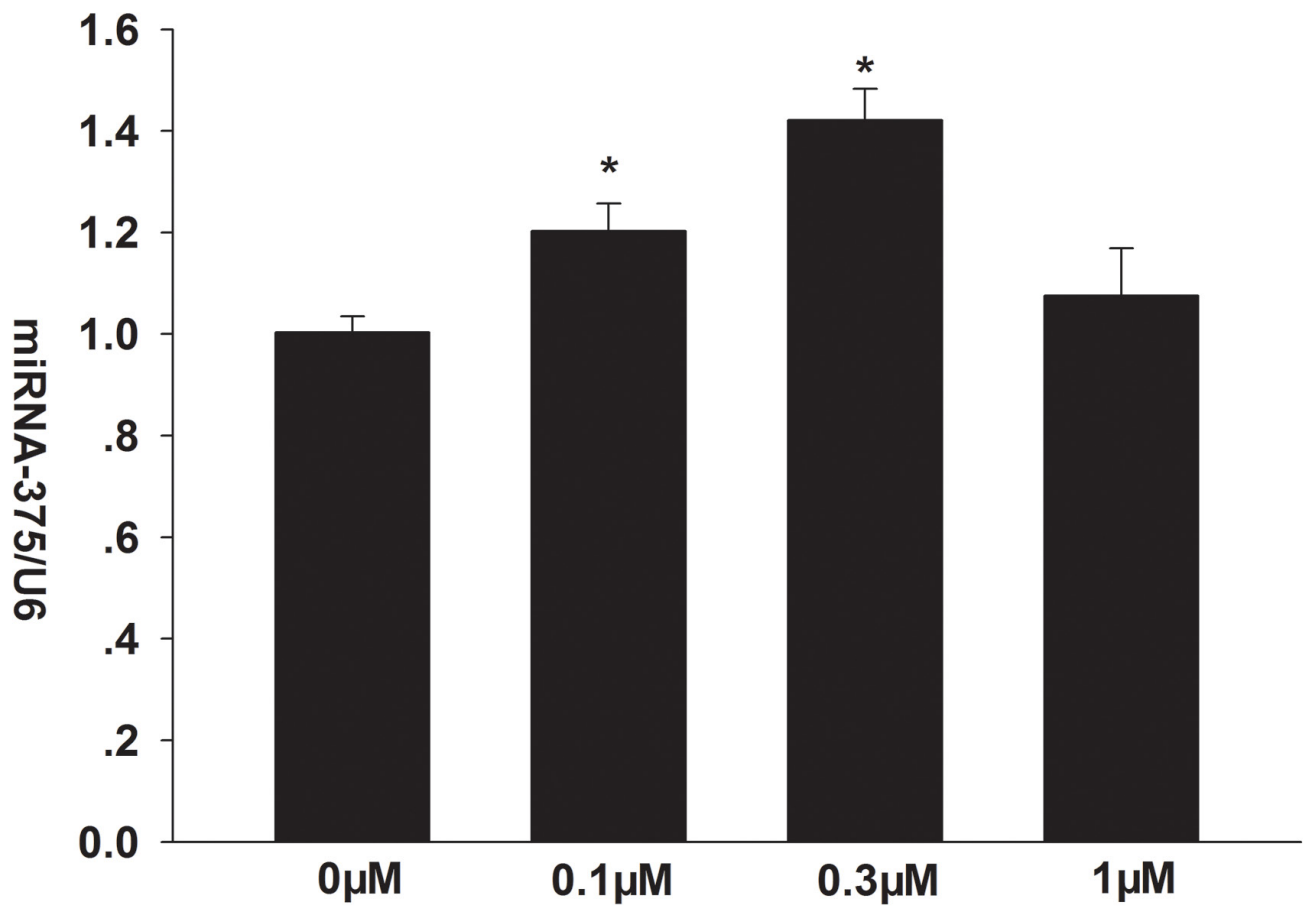
Effects of formononetin on the mRNA expression of *miR-375* in CNE2 cells *miR-375* mRNA expression was significantly upregulated by 0.1 and 0.3 μM formononetin. ^*^ = P < 0.05 vs control; n = 3.

Our RT-PCR analysis showed that 0.1 and 0.3 μM formononetin significantly increased miR-375 mRNA levels in CNE2 cells (0.1 μM formononetin, 1.203 ± 0.054%; 0.3 μM formononetin, 1.421 ± 0.062%; control, 1 ± 0.032%; p < 0.05) (Figure 3).

### Formononetin up-regulates ERα, p-ERK1/2, and bcl-2 expression and down-regulates PTEN expression in CNE2 cells

**Figure 4:**
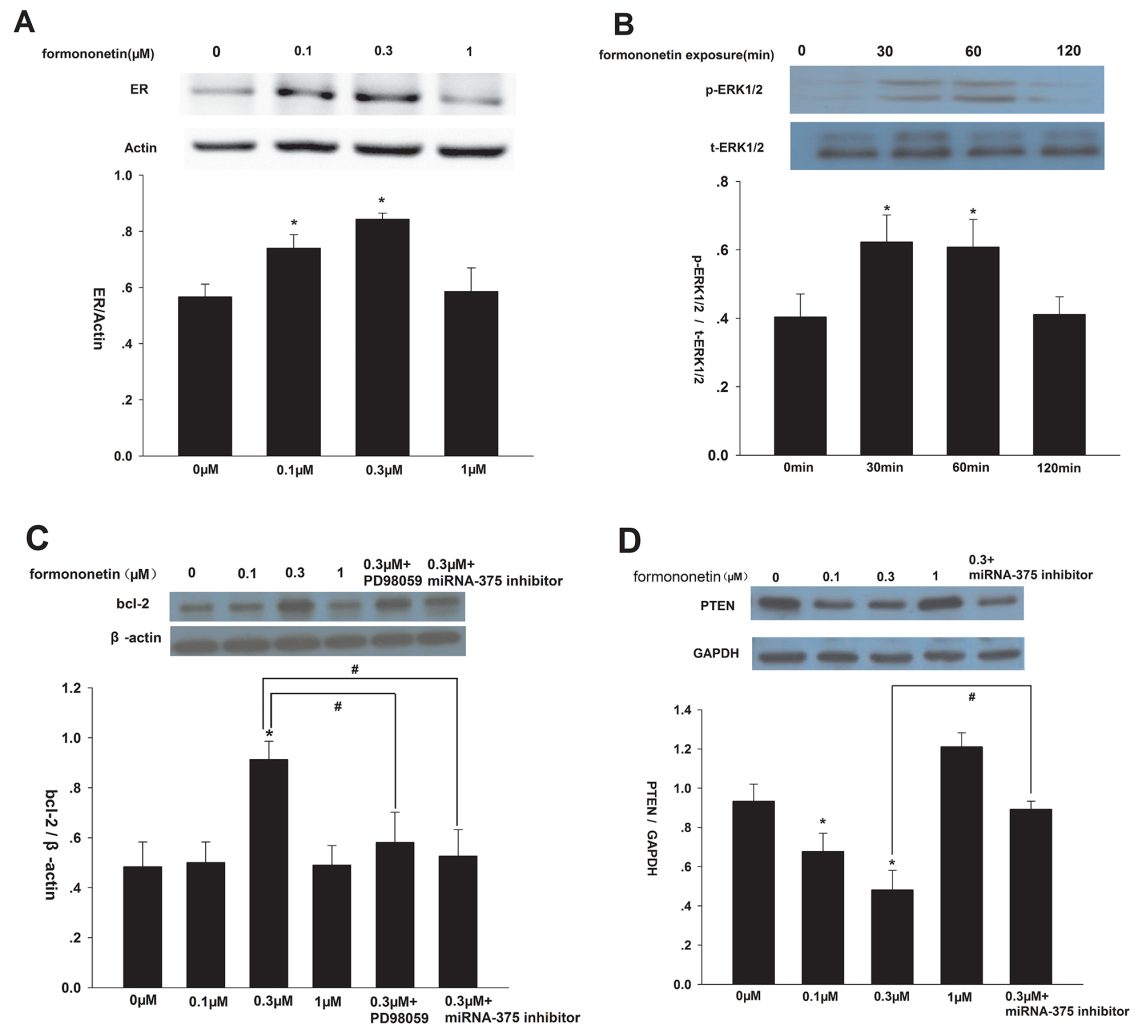
Effect of formononetin on ERα, p-ERK1/2, bcl-2 and PTEN protein expression in CNE2 cells **(A)** Effect of formononetin on ERα protein expression, low concentration of formononetin(up to 0.3 μM) increased the expression level of ERα protein. **(B)** Effect of formononetin on p-ERK1/2 protein expression, the rapid change of p-ERK1/2 by 0.3 μM formononetin at 0, 30, 60, 120 min was determined. **(C)** Effect of formononetin on bcl-2 protein expression. PD98059 and miR-375 inhibitor decreased the expression level of bcl-2 protein induced by 0.3 μM formononetin. **(D)** Effect of formononetin on PTEN protein expression.low concentration of formononetin (up to 0.3 μM) decreased the expression level of PTEN protein. MiR-375 inhibitor reversed the down-regulated effect. ^*^=P < 0.05 vs control; n=3. ^#^=P < 0.05 vs 0.3 μM formononetin group; n=3.

Compared to control, formononetin (0.1 and 0.3 μM) significantly increased ERα protein expression (p < 0.05), and ERα levels peaked in response to 0.3 μM formononetin (Figure 4A). However, there was no significant difference in ERα protein concentration in cells exposed to a high dose of formononetin (1 μM) (p > 0.05 *vs*. control). Furthermore, 0.3 μM formononetin treatment did not alter total ERK1/2 expression, but did induce a transient increase in p-ERK1/2 (Figure 4B). p-ERK1/2 levels significantly increased in cells incubated with 0.3 μM formononetin for 30 - 60 min, but then declined. In addition, formononetin (0.1 μM and 0.3 μM) dramatically up-regulated bcl-2 expression and down-regulated PTEN expression in CNE2 cells compared to the control (p < 0.05). This effect was reversed by the addition of either the MAP2K1 inhibitor, PD98059, or the miR-375 inhibitor (Figure 4C, 4D).

### Formononetin induces histological changes in the endometrium of OVX rats

**Figure 5:**
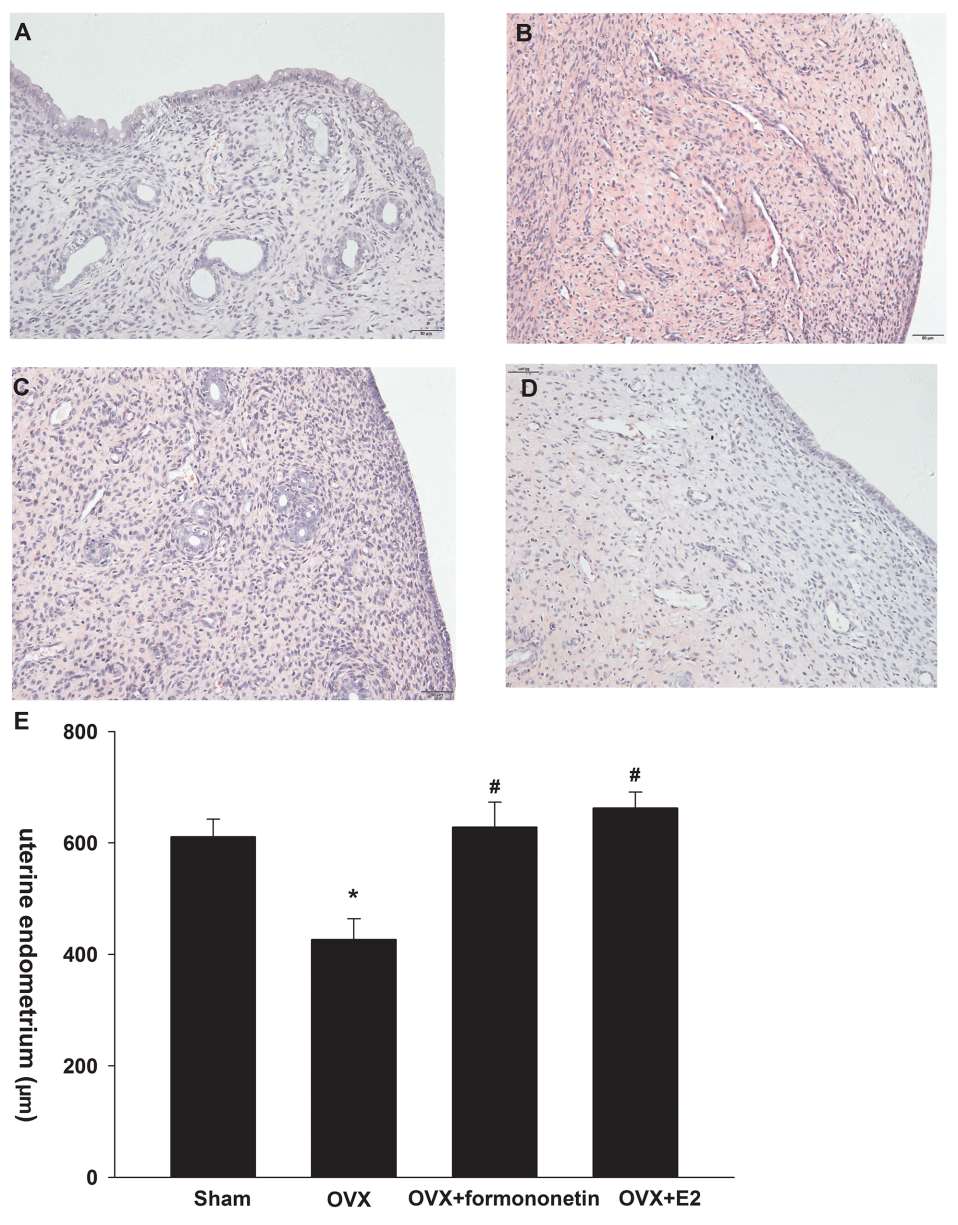
Effect of formononetin on the uterine endometrium of OVX rats I: Effect of formononetin on the form of uterine endometrium(HE: 200). **(A)** sham group; **(B)** OVX ; **(C)** OVX+8mg/Kg formononetin group and **(D)** OVX+20 μg/kg E2 group. II:Effect of formononetin on the thickness of uterine endometrium **(E)**. ^*^=P < 0.05 vs OVX; n=6. ^#^=P < 0.05 vs 0.3 μM formononetin group; n=6.

We also measured the *in vivo* effects of formononetin in the endometrium of OVX rats. As shown in Figure 5A-5D, endometrial epithelial cells were columnar shaped in the endometrium of sham operation controls. Flattened endometrial epithelial cells were detected in OVX rats. We observed columnar-shaped epithelial cells in OVX rats receiving either 8 mg/kg formononetin or 20 μg/kg E_2_. In addition, formononetin significantly increased the mean thickness of the endometrium compared to the OVX group (OVX, 426 ± 37 μm; formononetin, 628 ± 44 μm; p < 0.05) (Figure 5E). Similar results were obtained in the positive control group, in which OVX animals received an E_2_ injection. These findings indicate that formononetin stimulates endometrial growth in OVX rats.

### Formononetin inhibits ERα expression in uterine tissue of OVX rats

**Figure 6:**
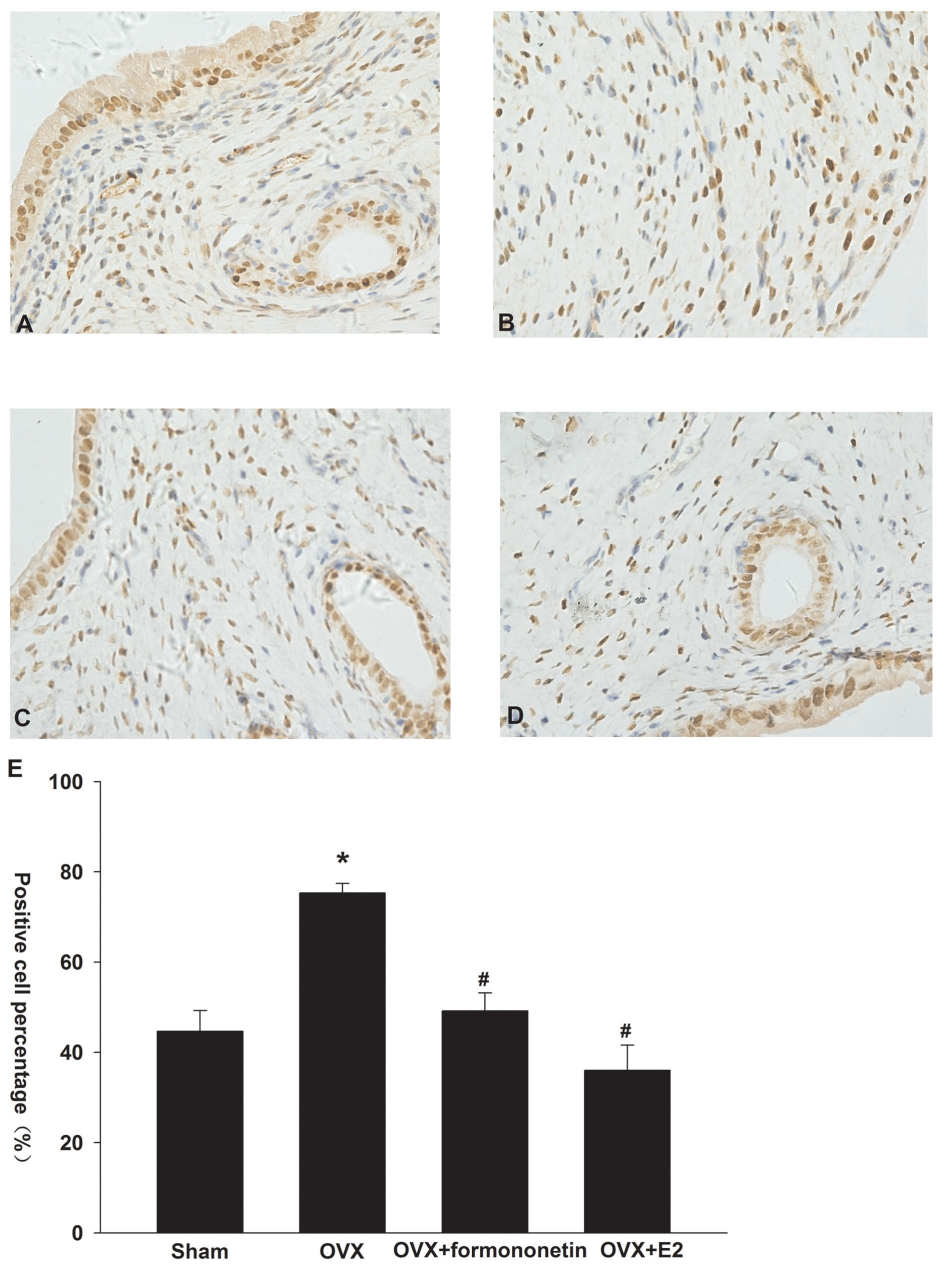
Effect of formononetin on the level of ERa protein in the uterine tissue of OVX rats **(A)** sham group; **(B)** control; **(C)** OVX+8mg/Kg formononetin group; **(D)** OVX+20 μg/kg E2 group and **(E)** the percentage of the positive cells. ^*^=P < 0.05 vs OVX; n=6. ^#^=P < 0.05 vs 0.3 μM formononetin group; n=6.

Immunohistochemical analysis demonstrated positive staining for ERα in the cellular membrane as well as in the cytoplasm of endometrial epithelial cells (Figure 6A-6D). OVX rats showed a significant up-regulation in ERα expression in the uterine tissues compared to the sham operation controls (the percentage of positive cells: sham, 44%; OVX, 75%; p < 0.05) (Figure 6E). Moreover, formononetin treatment significantly reduced the percentage of ERα-positive cells in OVX rats (49%). In the positive control group (E_2_ group), 36% cells expressed ERα in uterine tissues of OVX rats.

## DISCUSSION

Phytoestrogens, due to their natural source and low cost in synthesis and purification, have received extensive attention. Phytoestrogen has dual roles in mediating tumor cell growth, as it can both inhibit and promote growth. The proliferative effects of phytoestrogen appear to be associated with phytoestrogen concentrations. For instance, a high dose of phytoestrogen can suppress tumor cell growth [22, 23], but some phytoestrogens also exert proliferative effects at low concentrations [24–26]. In this study, we demonstrate that a low concentration of formononetin accelerates proliferation of ER-positive CNE2 cells *in vitro*.

ERs function as ligand-dependent transcriptional factors that regulate gene expression by enhancing transcription [27]. Increasing evidence indicates that ERs are expressed at an abnormal level in tumor tissues, which correlates with disease development and progression. It is suggested that ERs might serve as a novel target for the management and prevention of certain cancers [28]. In our previous study, we reported that formononetin inhibited growth in ER-positive cells, but not in ER-negative cells, implying that the growth mediated effects of formononetin depend on the binding and activation of ERs [29]. In the current study, we showed that a low dose of formononetin up-regulated ERα expression in CNE2 cells, indicating that the proliferative effects of formononetin on CNE2 cells are mediated, at least partially, through the ERα signaling pathway. These findings are in accordance with a previous report [30].

MiR-375 is located in a highly conserved intergenic region between the *cryba2* and *Ccdc108* genes [31]. Recently, miR-375 has been identified as an essential miRNA with the capacity to enhance ER signaling in cells [32]. Our recent report reveals a positive feedback loop between ERα and miR-375 in ER-positive breast cancer cells. Formononetin promotes the functional activation of ERα-miR-375. In this study, both ERα and miR-375 levels were up-regulated by formononetin. Moreover, the miR-375 inhibitor reversed the decreased percentage of late apoptotic cells and the protein expression of bcl-2 caused by formononetin. These findings confirmed that formononetin exerts a proliferative effect on CNE2 cells by activating miR-375. In addition, PTEN appears to be an essential target of miR-375 activity in formononetin-treated cells. PTEN is a classical tumor suppressor. Mutations and deletions of the PTEN gene have been identified in many types of cancers, including NPC [33]. PTEN plays an important role in the initiation and progression of NPC [34]. In this study, a low concentration of formononetin decreased PTEN protein expression, in turn leading to growth of CNE2 cells, which was effectively reversed by the miR-375 inhibitor.

A previous report indicates that PTEN acts as an upstream regulator of the ERK1/2 signaling pathway [35]. Overexpression of PTEN blocked the phosphorylation of ERK1/2 in MCF-7cells [36]. In accordance with this finding, Bouali *et al* also reported that reintroduction of PTEN significantly reduced p-ERK1/2 levels [37], implying that p-ERK1/2 may be negatively regulated by PTEN. Increased levels of p-ERK1/2 have been observed in pancreatic cancer cells [38]. In addition, inhibition of ERK1/2 triggers mitochondrial-mediated apoptosis in nasopharyngeal carcinoma cells [39], suggesting a positive correlation between p-ERK1/2 levels and cancer cell growth. Our previous study showed that treatment with 0.3 μM formononetin dramatically up-regulated the expression of p-ERK1/2 in cells. Additionally, the activation of ERK1/2 peaked within 10 to 20 min following formononetin treatment, but was decreased at 60 min after drug exposure [40]. Consistent with these findings, our current study shows that ERK1/2 activation peaked at 30 min following drug administration. Furthermore, formononetin resulted in sustained ERK activation for a duration of 12 - 48 h.

The MEK inhibitor PD98059 has been widely used to evaluate involvement of the ERK1/2 signaling pathway. PD98059 inhibits phosphorylation of MEK, inactivates the oncogene bcl-2, and ultimately increases apoptosis of cancer cells [41, 42]. Our results demonstrate that formononetin decreases the amount of late apoptotic cells and elevates bcl-2 protein expression, both of which were reversed by the addition of PD98059 and the miR-375 inhibitor. These findings imply that the proliferation of CNE2 cells induced by formononetin might be related to formononetin’s effect on miR-375-PTEN-ERK1/2-bcl-2 activation.

Our *in vivo* study indicates that formononetin dramatically increases cell proliferation in the endometrium in OVX rats. To elucidate the underlying mechanism of this observation, we examined ERα expression in rat uteri. In contrast to the results obtained in our *in vitro* cultured cells, we found that formononetin decreased uterine ERα expression. This discrepancy could be attributed to the complex *in vivo* environment, as formononetin may simultaneously activate multiple distinct pathways that function as networks [27]. The reduced ERα level in the presence of formononetin might be explained by a feedback loop that regulates ERα expression.

To our knowledge, this is the first study to show that the ERα-miR-375-PTEN-ERK1/2-bcl-2 pathway is involved in cell proliferation. The effects on proliferation may be attributed to the direct binding of formononetin to the ER, which then activates miR-375 and down-regulates PTEN expression. Furthermore, a decrease in PTEN expression drives ERK1/2 signaling and induces bcl-2 upregulation.

## MATERIALS AND METHODS

### Reagents

RPMI-1640 culture medium was purchased from Gibco-BRL, USA. Fetal bovine serum (FBS) was obtained from Hyclone, USA. The Supplementary File 1 was provided by Phytomarker Ltd., Tianjin, China. MAP2K1 inhibitor PD98059, miR-375 inhibitor and anti-bcl-2 antibody were purchased from Santa Cruz, CA, USA. Annexin V-FITC and propidium iodide (PI) were obtained from BD Biosciences, USA. Primary antibodies, including anti-p-ERK antibody, anti-t-ERK antibody and anti-ERα antibody were bought from Abcam, UK. Anti-β-actin and anti-GAPDH antibodies were obtained from Zhongshan Jinqiao, Beijing, China.

### Cell culture

The human nasopharyngeal carcinoma CNE2 cell line was bought from the Shanghai Institute of Cell Biology, the Chinese Academy of Sciences, Shanghai, China. Cells were cultured in RPMI-1640 medium containing 10% FBS, 100 kU/L penicillin and 100 mg/L streptomycin at 37°C in a 5% CO_2_ incubator.

### Drug treatment

Before experiments, cells were exposed to RPMI-1640 without phenol red supplementation for at least 4 days. Twenty-four hours prior to drug administration, culture medium was replaced with low-serum medium (RPMI-1640 containing 0.5% FBS). On the next day, cells were randomly divided into different groups, including control, 0.1 μM, 0.3 μM, 1 μM of formononetin, 0.3 μM formononetin + PD98059 or 0.3 μM formononetin + miR-375 inhibitor groups. In combined treatment groups, cells were pre-incubated with the MAP2K1 inhibitor PD98059 or the miR-375 inhibitor for 60 min, followed by co-administration of 0.3 μM formononetin and PD98059 or the miR-375 inhibitor. After 48 h of drug incubation, cells were used for analysis.

To determine p-ERK1/2protein expression, cells were treated with 0.3 μM formononetin for 0, 30, 60, and 120 min. To evaluate ERα protein expression, cells were exposed to 0, 0.1, 0.3, or 1 μM formononetin for 48 h. To measure bcl-2 and PTEN protein expression, cells were incubated with 0.1, 0.3 and 1 μM formononetin in the presence or absence of PD98059 or the miR-375 inhibitor for 48 h.

### Flow cytometric analysis

To analyze cell apoptosis, CNE2 cells were harvested and washed three times with ice-cold phosphate buffer solution (PBS). The cells were then stained with Annexin V-FITC and PI solution for 20 min at room temperature, followed by flow cytometric analysis. Cells that were positive for Annexin V-FITC and negative for PI were identified as early apoptotic cells. In contrast, cells positive for both Annexin V-FITC and PI were considered late apoptotic cells.

### Real-time PCR assay

CNE2 cells were treated with formononetin (0, 0.1, 0.3, and 1μM) for 48 h before harvesting. Then, total RNA was transcribed into cDNA using the Revert Aid ™ First Strand cDNA Synthesis Kit (Fermentas, Glen Burnie, MD, USA). mRNA expression was measured by quantitative real-time PCR using the ABI PRISM 7500 Sequence Detector System (Applied Biosystems, Carlsbad, CA). For product amplification, the following primer sequences of miR-375 were used: forward, 5′-CACAAAATTTGTTCGTTCGGCT-3′; reverse, 5′-GTGCAGGGTCCGAGGT-3′. The relative level of mRNA was normalized by U6 snRNA.

### Western-blot assay

CNE2 cells were collected and lysed in ice-cold lysis buffer. After centrifuging at 12,000 rpm for 15 min at 4°C, the supernatant was collected and stored at -80°C until use. The protein samples were separated by sodium dodecyl sulfate-polyacrylamide gel electrophoresis (SDS-PAGE) and transferred to polyvinylidene difluoride (PVDF) membranes (Millipore, Bedford, MA, USA). Membranes were blocked in 5% non-fat milk for 2 h at room temperature, followed by incubation with primary antibodies at 4°C overnight. The dilutions of primary antibodies were as follows: anti-bcl-2 (1:200), anti-p-ERK (1:400), anti-t-ERK (1:500), anti-ERα (1:400), anti-PTEN (1:1,000), anti-GAPDH (1:1,000), and anti-β-actin (1:1,500). After three washes with tris-buffered saline and Tween 20 (TBST) solution, samples were incubated with secondary antibody for 2 h. The immunoreactive bands were visualized using an electrochemiluminescence (ECL) reagent kit according to the manufacture’s instructions (Zhongshan Jinqiao, Beijing, China).

### Animals and experimental groups

Female Sprague Dawley rats, weighing 250 - 270 g, were obtained from Hunan SJA Laboratory Animal Co., Ltd. Animals were housed in a specific pathogen-free room at a constant temperature of 18 - 23°C and 55 - 65% humidity with a 12 h /12 h light / dark cycle.

Rats were anesthetized by intraperitoneal (i.p) injection of 10% chloral hydrate. To generate the OVX animal model, both ovaries were removed from female rats. Seven days after surgery, animals were divided into four groups: Sham (n = 6), OVX (n = 6), OVX + formononetin (n = 6), and OVX + E_2_ (n = 6). In the OVX + formononetin and OVX + E_2_ groups, animals were given daily i.p. 8 mg/kg·d formononetin or 20 μg/kg·d E_2_. Rats in the Sham and OVX groups received daily i.p injections of normal saline and vehicle solution, respectively. After 30 days of drug administration, the uteri of rats were removed, fixed in 10% neutral formalin, dehydrated, embedded in paraffin, and sectioned into 5-μm-thick slices. Some sections were stained with hematoxylin and eosin (H & E) for histological examination and some were stained with specific antibodies for immunohistochemical analysis.

### Immunohistochemistry

After deparaffinization in xylene and hydration through graded alcohols, sections were incubated with 3% H_2_O_2_ for 10 min, followed by three PBS washes. Antigen retrieval was conducted by microwave treatment of samples in citrate buffer (pH 6.0). Sections were then incubated with the primary antibodies (1:500) for 1 h at 37°C. After washing in PBS, samples were probed with secondary antibody using a *PV-9000* polymer detection kit (Zhongshan, China). Immunoreactivity was visualized using 3, 3-diaminobenzidine (DAB). Samples were counterstained with hematoxylin and observed under light microscopy. For each slide, 100 cells were randomly selected, and the number of immunopositive cells was counted. The percentage of immunopositive cells was calculated from three slides for each group.

### Statistical analysis

Data are presented as mean ± standard error (SE). Statistical analysis was performed using SPSS 17.0 software. Student’s t-test was used for comparison between two groups. A p value < 0.05 was considered statistically significant.

## SUPPLEMENTARY MATERIALS





## Abbreviations

ER: estrogen receptor
OVX: ovariectomized
HE: hematoxylin and eosin
FBS: fetal bovine serum
DAB: 3,3-diaminobenzidine
MAPK: mitogen-activated protein kinase
PTEN: protein-phosphatase and tensin homologue
