# Supplementary material for: Low concentration of formononetin promotes proliferation of estrogen receptor-positive cells through an ERα-miR-375-PTEN-ERK1/2-bcl-2 pathway
Source: Oncotarget. 2017 Oct 19;8(59):100045–55. doi: 10.18632/oncotarget.21923 (PMC5725001; doi:10.18632/oncotarget.21923)
Supplement: Supplementary file 1 [file oncotarget-08-100045-s001.pdf]

Low concentration of formononetin promotes proliferation of estrogen receptor-positive cells through an ERα-miR-375-PTEN-ERK1/2-bcl-2 pathway

SUPPLEMENTARY MATERIALS

Supplementary File 1: The heading for Supplementary File 1 is HPLC chromatogram for determination of formononetin purity.

See Supplementary File 1
