# Supplementary material for: Low concentration of formononetin promotes proliferation of estrogen receptor-positive cells through an ERα-miR-375-PTEN-ERK1/2-bcl-2 pathway
Source: Oncotarget. 2017 Oct 19;8(59):100045–55. doi: 10.18632/oncotarget.21923 (PMC5725001; doi:10.18632/oncotarget.21923)
Supplement: Supplementary file 2 [file oncotarget-08-100045-s002.doc]

**Product ID:**

**Marker For**: **Name:**

**CAS Number:**

PCM-AM-006

*Astragalus membranaceus (Fisch.) Bunge*

Formononetin

485-72-3

**Structure:**


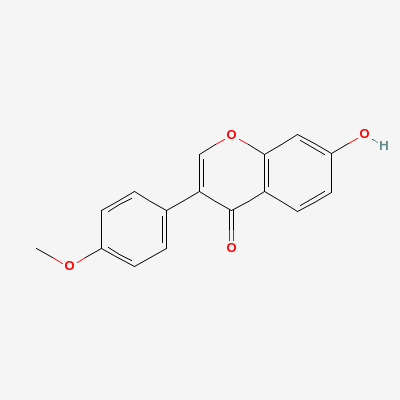

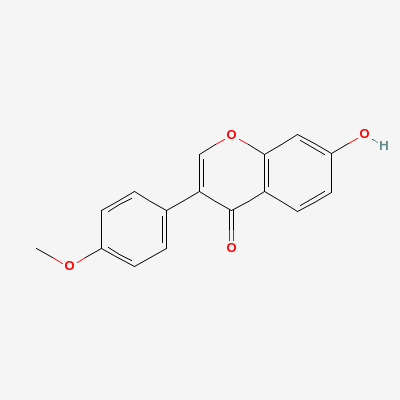
 [
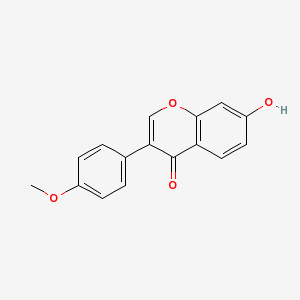
](javascript:%20void%20window.open('../image/structurefly.cgi?cid=5280378&width=400&height=400',%20'StructureFly',%20'resizable=yes,%20scrollbars=yes,%20WIDTH=620,%20HEIGHT%20=%20620')) [
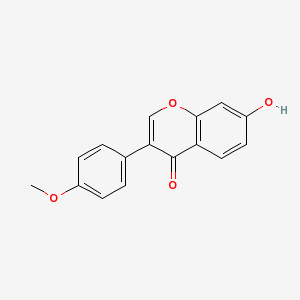
](javascript:%20void%20window.open('../image/structurefly.cgi?cid=5280378&width=400&height=400',%20'StructureFly',%20'resizable=yes,%20scrollbars=yes,%20WIDTH=620,%20HEIGHT%20=%20620'))

**Molecular Formula:**

**Molecular Wieght**:

**Melting Point:**

**Solubility:**

**Character:**

C_16_H_12_O_4_

268.26408 [g/mol]

258-259℃

Respectively soluble in Chloroform, Alcohol, Methanol, Acetone. Insoluble in Water.

White Powder

**Storage Condition:**

**Package:**

**Storage:**

Brown glass bottle

5℃ with good seal. Vacuum drying with P2O5 for 12 hours before using.

**Analytical Results:**

| Test | Specificaiton | Result |
| --- | --- | --- |
| HPLC | NA | 99.32% |
| Mass Spectrum | Conforms | Conform |

**Analysis Conditions:**

Intrument:

Column:

Column Temperature:

Mobile Phase:

Flow Rate:

Detection:

Agilent 1100 Series LC

Agilent C-18 250*4.6mm 5μ

30℃

Methanol – Water 55-45

1.0ml/min

254nm

**HPLC Chormatogram of Formononetin:**

**:**

**:**

**:**
